# Bariatric surgery as bridging therapy to kidney transplantation

**DOI:** 10.1007/s00467-025-06661-0

**Published:** 2025-03-18

**Authors:** Anke Raaijmakers, Belinda Dooley, Blake Sandery, Swasti Chaturvedi, Renee Le Jambre, David Links, Sean E. Kennedy

**Affiliations:** 1 Department of Paediatric Nephrology, Sydney Children’s Hospital Randwick, Randwick, Australia; 2 School of Women’s and Children’s Health, University of New South Wales, Randwick, Australia; 3 Department of Pediatrics, Division of Nephrology, University of British Columbia, Vancouver, Canada; 4 Division of Nephrology, British Columbia Children’s Hospital, Vancouver, Canada; 5 College of Medicine and Public Health, Flinders University, Adelaide, Australia; 6 Department of Nutrition and Dietetics, Sydney Children’s Hospital Randwick, Randwick, Australia; 7 Department of Surgery, Prince of Wales Hospital, Randwick, Australia

**Keywords:** Kidney failure, Chronic kidney disease, Obesity, Bariatric surgery

## Abstract

The prevalence of obesity in adolescents is rising, including in those with kidney failure. Obesity increases the risk of complications during dialysis and may be associated with poorer outcomes after transplantation. Bariatric surgery has been found safe and effective in adults on dialysis. This Clinical Insights is about a 14-year-old female with kidney failure and obesity. She was initially managed on peritoneal dialysis but subsequently gained further weight. Multiple interventions for weight loss were unsuccessful, including the switch to haemodialysis. The patient underwent laparoscopic sleeve gastrectomy after extensive multidisciplinary assessment. She lost > 30 kg over 6 months (BMI decreased from 48 to 32 kg/m^2^) which made it possible for her to be activated on the deceased donor kidney transplant waiting list. Managing weight gain in patients on peritoneal dialysis is important, especially in obese adolescents. Bariatric surgery should be considered early where morbid obesity is a major impediment to listing for kidney transplantation.

## Introduction

Obesity in children and adolescents is an emerging problem, including in adolescents with kidney failure [1]. Prevalences for overweight (body mass index, BMI > 25 kg/m^2^) and obesity (BMI > 30 kg/m^2^) in this group are ~ 20% and ~ 12%, respectively [2]. Obesity may increase the risk of complications during dialysis and is associated with poorer short- and long-term outcomes after transplantation [1–3]. Bariatric surgery has been used in adults on dialysis and is considered safe and effective as bridging therapy to kidney transplantation [3].

Peritoneal dialysis can cause significant glucose absorption with additional calories up to 1200 kcal/session [4, 5] and this needs to be considered when choosing dialysis modality in patients with pre-existing obesity. Bariatric surgery should be considered early where morbid obesity is an obstacle to listing for kidney transplantation. Moreover, conservative treatment failure puts them at risk of prolonged dialysis. In this clinical insight article, we report on successful bariatric surgery in an adolescent on haemodialysis after failure of multiple conservative weight loss interventions.

## Case report

A 14-year-old female presented to our hospital with kidney failure due to reflux nephropathy. She had a BMI of 32 kg/m^2^ at the time of presentation. She was started on peritoneal dialysis. She had a background of bilateral vesicoureteral reflux disease and recurrent urinary tract infections in the first years of her life. The family changed home address several times and she was lost to follow up.

**Fig. 1:**
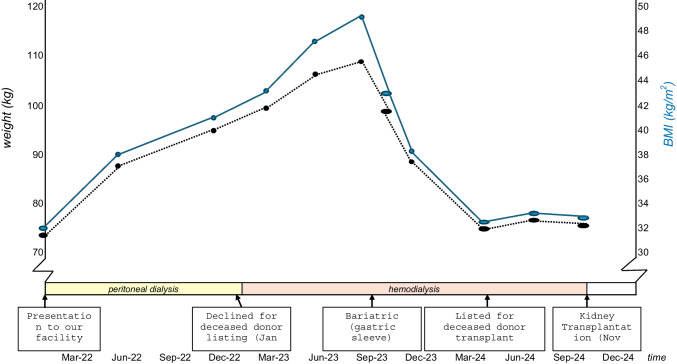
Weight and management trajectory

Whilst on peritoneal dialysis for 12 months, her weight increased by > 20 kg and her BMI increased to > 40 kg/m^2^ (Fig. 1). The patient was considered for listing for a deceased donor transplant and declined due to high BMI (42) and central obesity. An echocardiographic screen did not reveal any strain on the heart nor was she on antihypertensives. She was switched to haemodialysis, however, gained further weight. Further investigations (lipid studies, glucose and hormone work-up, thyroid function, etc.) could not reveal any reversible cause of excessive weight gain (HOMA-IR score of 0.3). Multiple interventions for weight loss (exercise induction, diet modifications, multidisciplinary weight management clinic, family training, weekly dietician review, etc.) were unsuccessful and after multidisciplinary assessment, the patient was deemed suitable for bariatric surgery (laparoscopic sleeve gastrectomy). Before surgery, she followed a supervised very low-calorie diet (VLCD) which consisted of commercially available VLCD shakes (Optifast^©^) and non-starchy vegetables for 4 weeks. Shakes had to be paired with a phosphate binder and serum electrolytes, calcium and phosphate were monitored weekly.

In September 2023, she underwent surgery and continued thrice weekly haemodialysis. After bariatric surgery, her diet was graded up as per bariatric surgery recommendations. She had clear fluids for several days, then free fluids for 2 weeks, followed by puree for 2 weeks, a soft diet for 2 weeks and then finally back to a normal diet texture. Her diet was adapted to be low potassium and low phosphate whilst also complying with her fluid restriction. She continued with VLCD shakes and a phosphate binder with additional kidney dietary supplements to meet her suggested daily intake (SDI) for protein whilst on the puree phase. A vitamin B supplement was commenced to account for decreased absorption of B vitamins after bariatric surgery. We did not use any commercial bariatric surgery multivitamins due to the high vitamin A content. She also temporarily commenced a calcium supplement to meet SDI whilst her diet had very limited variety.

Monitoring of serum B12, folate, zinc, selenium and fat-soluble vitamins, occurred at 1 month, 3 months, and 6 months. Regular anthropometric measures including weight, height, and waist circumference were monitored pre- and post-surgery (Fig. 1). The patient lost over 30 kg 6 months post-surgery, and the weight loss enabled her to be activated on the deceased donor list for kidney transplantation. She was successfully transplanted after 7 months of listing.

At the time of transplant, we considered weaning her steroids more quickly than our regular protocol (mycophenolate, tacrolimus and steroids with a start dose 2 mg/kg/day with weekly 2.5–5 mg wean). She received a high dose intraoperatively (methylprednisolone 500 mg) prior to clamp release and 80 mg daily from Day 1. However, she was not weaned for the first few weeks as we failed to reach therapeutic tacrolimus levels. With the introduction of diltiazem as CYP3A4 inhibitor, we managed to successfully wean her steroids after 3 weeks. Although she had a single high fasting glucose (5.9 mmol/L) this normalised afterwards (4.2 mmol/L).

## Discussion

This Clinical Insights case report discusses the safe and successful use of paediatric bariatric surgery as bridging therapy to kidney transplantation. Our patient lost >30 kg post-surgery which made it possible to list her on the waitlist for a deceased donor transplant kidney. To the best of our knowledge, this was the first reported adolescent haemodialysis patient who received bariatric surgery to enable listing for kidney transplantation.

Morbid obesity and central adiposity can make kidney transplantation a technically much more challenging operation due to difficulty accessing the surgical field and increases the risk of complications. Bariatric surgery has been used successfully as bridging therapy in adults [3]. Obesity rates are rising worldwide, and they might be even more prevalent in children with chronic kidney disease (CKD) and kidney failure [1, 2]. Although clear adverse effects of obesity after kidney transplantation are lacking [1], the evidence of adverse cardiovascular effects is obvious [2]. Unfortunately, whilst these adolescents face increased cardiovascular risks including metabolic syndrome, they also experience barriers progressing to kidney transplantation [2]. Altemose et al. recently described the barriers that paediatric obese patients with CKD and kidney failure face in their way towards kidney transplantation [1]. The most important issue was the inability to meet the BMI recommendations for transplant [3]. However, we must acknowledge that the decision not to list our patient was a centre decision and might not be the same in other centres around the world. The KDIGO guideline [3] reports that patients with a BMI >30 convey an increased risk of death, delayed graft function, acute rejection, as well as wound dehiscence and prolonged hospital stay. They further state that ‘transplantation in patients with a BMI >40 kg/m^2^ should be approached with caution as patients need to understand the increased risk of post-operative complications’ [3], but there is no robust evidence to support the exclusion of children from transplant listing solely based on BMI. Interventions should be considered including bariatric surgery [3].

The long-term consequences of bariatric surgery in adolescents with kidney disease are unknown. Although adult data report successful bridging to transplantation [3], follow-up data in adolescents are not available. Importantly, adolescents need to be deemed suitable for bariatric surgery including a thorough developmental/mental health assessment [1, 3]. We would like to highlight that our patient ‘only’ prolonged her dialysis for 7 months after her bariatric surgery, this additional time on dialysis might have put her at increased risk of infection and long-term cardiovascular effects on her vessels.

Glucose absorption in patients on peritoneal dialysis varies depending on the formula used, the mode of dialysis and membrane characteristics [4], but can be significant. Kotla et al. recently proposed a model to estimate glucose absorption in adult patients on peritoneal dialysis, which seems to be around 100 g/day corresponding with ~ 400 kcal per day. In paediatric patients on automated short cycle peritoneal dialysis, we estimate that the additional calories might be even higher (up to 1200 kcal/session) [5]. However, our patient continued to gain weight after switching to haemodialysis, so we know there must be additional factors involved. We suggest considering the importance of significant weight gain in patients on peritoneal dialysis (due to glucose absorption [5]), especially in obese adolescents.

In conclusion, bariatric surgery should be considered in obese adolescents with kidney failure on dialysis as bridging therapy to kidney transplant. Patient work-up, perioperative management and long-term monitoring of weight loss and nutritional needs should be managed by an experienced multidisciplinary team. We have shown that significant weight loss can be achieved in an adolescent with kidney failure.

## Summary

### What is new?

- Decision regarding dialysis modality should consider the risk of weight gain on peritoneal dialysis, especially in obese adolescents. Bariatric surgery should be contemplated early where morbid obesity is an obstacle to listing for kidney transplantation.

## Data Availability

All data were included in the manuscript.

## Abbreviations

BMI: Body mass index
CKD: Chronic kidney disease
